# Postoperative wound assessment in cattle: How reliable is the back hand palpation?

**DOI:** 10.1186/s13620-021-00195-1

**Published:** 2021-06-16

**Authors:** Ioannis Proios, Marian Kusenda, Christian Seiler, Carsten Siewert, Hermann Seifert, Martin Kaske

**Affiliations:** 1 Clinic for Cattle, University of Veterinary Medicine Hannover, Foundation, Hannover, Germany; 2 Nord-Ostsee Tierärzte, Veterinary Practice, Schafflund, Germany; 3 Institute for General Radiology and Medical Physics, University of Veterinary Medicine Hannover, Foundation, Hannover, Germany; 4 Department for Farm Animals, Vetsuisse Faculty, Zurich, Switzerland

**Keywords:** Cows, Infrared imaging, Laparotomy, Palpation, Skin temperature, Thermography, Wound healing

## Abstract

**Background:**

As part of clinical wound assessment in bovine surgery, discrepancies in skin temperature are evaluated by placing the back of the hand on the area to be examined. Generally, an increased skin temperature at the wound site for a prolonged period is considered as an indicator of impaired wound healing.

The aim of this study was to verify the reliability of palpation under bovine practice conditions using laparotomy as an example. Fourteen cows (German Holstein) with a left displacement of the abomasum (LDA) without other severe concurrent diseases were examined preoperatively and once daily for ten days after surgery. The skin temperature of the wound site in the right flank was assessed by palpation, followed by thermographic evaluation using an infrared camera after a 45-min acclimatisation period, under standardised conditions in a closed examination room daily for 10 days.

**Results:**

All the incisions healed without clinical detectable perturbances. The ambient temperature range during the study period was 7.8 − 24.1 °C. Two groups were retrospectively defined according to the ambient temperature: high ambient temperature (HT group; median: 20.2 °C 25/75 quartile: 18.5 °C / 21.7 °C; *n* = 6) and low ambient temperature (LT group; 10.8 °C; 9.4 °C / 12.8 °C; *n* = 8). The temperature differences (Δϑ) between the mean skin temperature of the wound site and a defined reference area cranial to the wound were assessed. A significant negative correlation was found between the ambient temperature (ϑ_Amb_) and Δϑ (*r*=-0.51; *P* < 0.001). The Δϑ was postoperatively higher in the cows in the LT group (median of the individual animals 0.8–2.5 °C) than in the HT group (0.1–0.5 °C; *P* < 0.05). In contrast to the thermographic findings, manual palpation rarely detected local hyperthermia (> 1 °C) at the wound site (sensitivity 0.20; specificity 0.96).

**Conclusions:**

The infrared thermography provides a more reliable assessment of temperature changes at the wound site in comparison to manual palpation. The ambient temperature markedly affects the extent of local hyperthermia at the wound site.

## Background

Standing flank laparotomy is a commonly used procedure in cows. Disturbed postoperative wound healing can delay the recovery of cows and thus increase treatment costs. The wound healing process is usually assessed clinically by inspection and palpation. Important diagnostic evidence regarding wound healing could be provided by determining several clinical parameters, and the surface temperature of the wound site is one of them [1]. Although an inflammatory phase occurs during the physiological course of wound healing, a profoundly increased skin temperature at the wound edges for a longer period of time is considered as a sign of impaired wound healing [2, 3]. Thus, during palpatory examination of cows, deviations in skin temperature are at least qualitatively checked by using the back of the examiner’s hand [4]. In human medicine, on the other hand, the palpation assessment of skin temperature appears to be subjective and quite unreliable [5]. Non-invasive infrared (IR) thermography is suitable for an objective and quantitative assessment of the skin temperature in the wound area [6–8], thus providing an indication of circulatory disorders, inflammation and impaired wound healing.

The aim of the present study was to investigate the reliability of palpation in estimating skin temperature under working conditions. Therefore, we compared the results of the palpation assessment of the skin temperature with the IR thermography measurements during the postoperative period of cows undergoing right flank laparotomy and omentopexy.

## Methods

### Experimental animals

Fourteen dairy cows (German Holstein) admitted to the Clinic for Cattle of the University of Veterinary Medicine Hannover Foundation, Hannover, Germany due to a left-sided displacement of the abomasum were included in this prospective study. Based on a comprehensive clinical investigation, cows did not suffer from a further accompanying disease.

### Timeline of interventions

As part of the usual preoperative preparation [9], the right flank was first washed with lukewarm water and soap in a preparation room, shaved extensively and wiped dry with a towel. The cows were then taken to the adjacent and unheated examination room.

All cows were administered procaine penicillin G once daily for four days, beginning the day of surgery (30,000 IU/kg; s.c.; Procain-Penicillin G ad us. vet. ®; aniMedica GmbH, Senden-Bosensell, Germany). Meloxicam was also administered preoperatively (0.5 mg/kg; s.c.; Metacam®; Boehringer Ingelheim Pharma GmbH & Co. KG, Ingelheim, Germany).

After the preoperative measurements, the animal was taken to the operating theatre.

The shaved right flank was cleansed with 70 % ethyl alcohol and povidone iodine solution (Vet-Sept® 10 %, Albrecht GmbH, Aulendorf, Germany). The distal paravertebral block was combined with a linear infiltration of the abdominal wall at the incision sites (laparotomy and omentopexy wound). For the locoregional flank anaesthesia 180 ml procaine hydrochloride with epinephrine were used (Isocain® 2 %; Selectavet Dr. Otto Fischer, Weyarn-Holzolling, Germany). The flank was then cleansed once again with povidone iodine solution.

The abomasal displacement was corrected through a right flank approach and an omentopexy performed using a button-plate combination [10, 11]. The abdominal wall was closed in three layers in accordance with the standard closure technique of our clinic. The skin and the external oblique abdominal muscle were closed together with five modified interrupted vertical Donati sutures (Silk® USP 8; SMI AG, St. Vith, Belgium).

The surgical wound was treated with chlortetracycline spray (Chlortetracycline-Spray®; Novartis Tiergesundheit GmbH, Munich, Germany). The duration of the operation and the length of the incision were documented for each case. In order to minimise the surgeons’ interindividual impact on wound healing, the cows were operated exclusively by two experienced veterinary clinic assistants.

 Following surgery, each animal received 200 ml propylene glycol orally twice a day for five days. Clinical examination and the specific wound assessment of the incision by palpation and IR image were performed daily from day 1 to 10 *post operationem* (p. op) at 10:00 on each day in the same examination room, which was protected from sunlight and draughts. Daily examinations were performed always by the same person (I.P.). After being discharged from the clinic on the 10th day after surgery, information concerning the animal’s performance and the aspect of the surgical wound was investigated through a telephone interview with the animal’s owner between day 30 and 60 after surgery.

### Assessment of data

The ambient temperature and humidity in the examination room were documented using a digital thermohygrometer (GMH®3330 Greisinger electronic GmbH, Regenstauf, Germany).

**Fig. 1:**
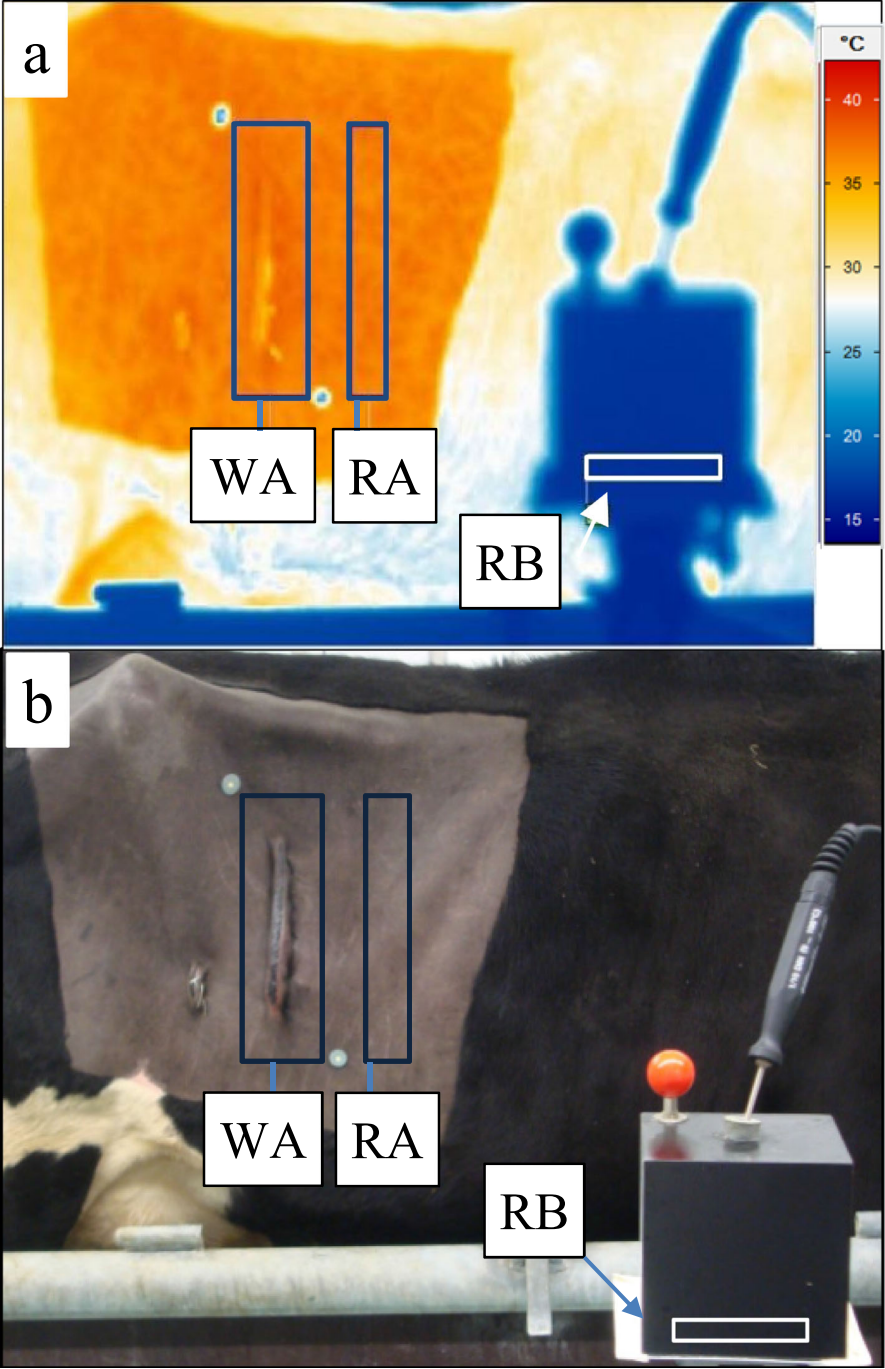
IR image presented with the software IRBIS®3 plus (**a**) and digital picture (**b**) of the right flank of cow postoperatively. Schematic illustration of the defined skin areas: WA = wound area, RA = reference area cranial of the laparotomy wound, RB = measured area of the reference body

Inspection of the wound was carried out to assess any swelling or exudation of the incision. Palpation focused on skin temperature by placing the back (dorsal surface) of the hand alternately in the area next to the incision (wound area) and about 20–30 cm cranial to the wound on the reference area (Fig. 1). Thus, it was palpatorically estimated whether the skin of the wound area was colder, normal or warmer in comparison to the reference area. Furthermore, the consistency of the wound area (soft, firm, rubbery, fluctuating or emphysematous) and its sensitivity to compression were determined through palpation (according to the defensive response of the cow). All these aspects of clinical examination of the wound area were evaluated with a grading system (Table 1).

**Table 1:** Grading system for clinical wound assessment

	clinical wound assesment score			
	0	1	2	3
Swelling	none	slight (< 1/3 the periwound surface)	moderate (1/3 − 2/3 the periwound surface)	severe > 2/3 the periwound surface
Consistency	soft	firm-rubbery	emphysematous	fluctuating
Exudation	none	slightly odourless discharge	a great amount of odourless discharge	malodorous discharge
Sensitivity to compression	none	slight	moderate	severe
Palpation (skin temperature)	normal	colder or warmer in comparison to the reference area		

**Fig. 2:**
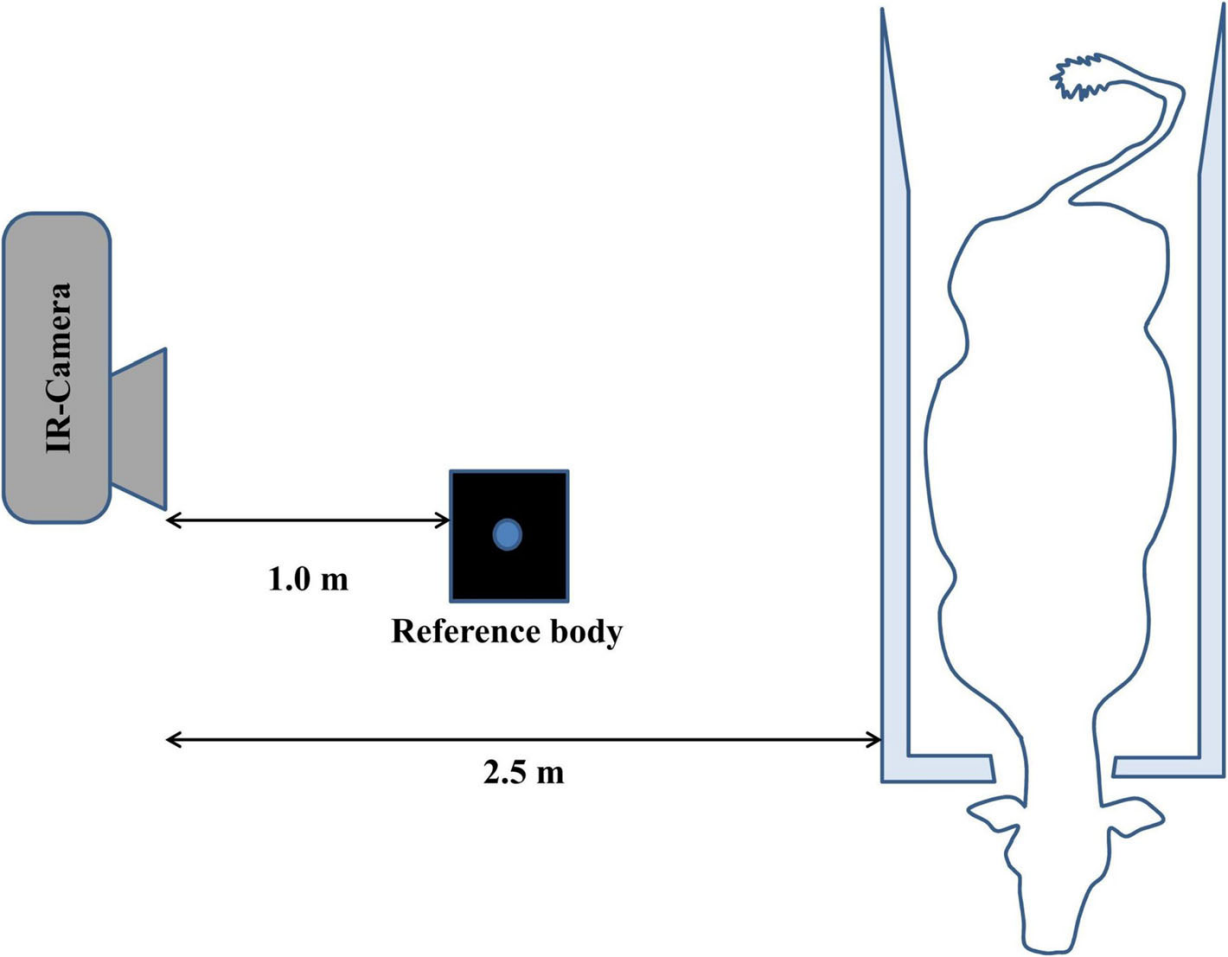
Schematic illustration of the measuring set-up for infrared thermography. The reference body was continuously positioned at a specific point in front of the IR camera and in this way it appeared in each IR image of the right flank of the cow

To guarantee a standardised vertical IR image in relation to the sagittal surface of the animals, the cows were fixed in a stand with a front interception lock during the examination. The distance between the IR camera and the right flank was constantly 2.5 m, and the distance of the IR camera from the ground 1.2 m (Fig. 2).

The daily postoperative IR image was taken after a 45-min acclimatisation period using an IR camera (IR FlexCam®R2, GORATEC Technology GmbH & Co. KG, Erding, Germany) with the entire right flank of the cow being mapped. The IR camera operated in a spectral range of 8–12 μm. The image matrix of the microbolometer sensor consisted of 160 × 120 pixels (interpolated internally to 320 × 240 pixels). The thermal resolution was in accordance with manufacturer’s specifications of 70 mK. The accuracy of the absolute temperature measurement of the IR camera in accordance with the manufacturer’s specifications was ± 2 °C or ± 2 % [12]. The IR camera was switched on ten minutes prior to the shooting. The IR camera was auto-calibrated immediately before each image to eliminate homogeneity errors. The emissivity ε of the skin was set at 0.98, as previously reported in the literature [13, 14]. The absolute temperature measurements were subsequently adjusted using a reference body, adapted to the room temperature displayed on every IR image (Fig. 1). The reference body used in this study was a cube of copper sheet filled with water with a matt black front side coating with an integrated stirrer (Leslie’s Cube, Cornelsen Experimenta GmbH, Berlin, Germany). The reference body was positioned on a tripod (1.15 m above the ground) and at a distance of one metre from the IR camera (Fig. 2). The reference body was left in the examination room throughout the study to be in constant thermal equilibrium with the ambient temperature. By moving the stirrer gently up and down, an almost equal temperature distribution of the water inside the cube and an even heat radiation from the black front side were achieved. The temperature of the reference body was measured and recorded during the investigation using a precision thermometer (GMH®370; GREISINGER electronic GmbH). For correcting the absolute temperatures of each IR image, the difference in temperature of the reference body measured by the IR camera and the temperature measured with the thermometer was calculated for each image. The result was subtracted from the corresponding IR image.

The IR images were evaluated using the Region of Interest (ROI) analysis method to minimise the time-related drift error of the temperature measurement [15]. For this purpose, two easy-to-locate skin areas were determined: the wound area (WA) as the “target area” and the reference area (RA). The temperature differences (Δϑ) were calculated between the mean temperatures of the WA (ϑ_WA_) and the RA (ϑ_RA_). An accurate outline of the wound area in the right flank was achieved by two metallic rings (Ø 2.5 cm), which were temporarily attached caudodorsally and cranioventrally to the incision line at defined points with double-sided adhesive tape. The exact positions were determined using a right-angled triangle template (hypotenuse 9.3 cm). These rings were clearly visible on the IR images and subsequently enabled a precise and reproducible determination of the ROIs (Fig. 1) and their evaluation. The thermography software IRBIS® 3 Plus (Infratec GmbH, Dresden, Germany) automatically calculated the mean temperatures of the defined skin areas of the WA (ϑ_WA_) and the RA (ϑ_RA_) on every IR image.

## Evaluation of data

The results for each parameter were tested for normal distribution using the Kolmogorov-Smirnov test. As the results were mostly not normally distributed, these were given as medians with 25 and 75 % quartiles. The Wilcoxon (signed rank) test was used to compare the temperature measurements between different days (before and/or after the surgery), as well as the milk yield preoperatively and at day 10 after surgery. Correlations were calculated using the Spearman’s rank correlation coefficient. The data were considered statistically significant when the probability of error was below 5 % (*P* < 0.05). The sensitivity and specificity of the manual palpation detection of local hyperthermia of the skin were evaluated in relation to the objective IR thermographic measurements Δϑ. Sensitivity was defined as the ratio of the number of wounds with increased palpatory warmth to the number of wounds with increased temperature (based on IR thermographic measurements; temperature difference > 1 °C).

The specificity was defined as the ratio of the number of wounds not found to be warm by palpation to the number of wounds that were not warm, based on IR thermographic measurements (temperature difference < 1 °C). Based on studies in equine medicine, where a temperature difference of up to 1 °C between two anatomically symmetrical skin areas is considered normal [16], the crucial temperature difference was set at 1 °C in our study.

The cows were retrospectively assigned to two groups according to the ambient temperature (ϑ_Amb_) using the Cluster Procedure with SAS® (Version 9.1, Statistical Analysis System Institute INC., Cary, NC, USA).

## Results

On the day of surgery as well as during the postoperative period (day 1 to 10), all cows showed an undisturbed general condition and physiological rectal temperature (38.6 °C; 38.4 °C / 39.0 °C; median; Q1 / Q3). The milk yield increased from the day of surgery (16.0 l; 10.3 l / 17.8 l; median; Q1 / Q3) until discharge on day 10 (22.0 l; 23.5 l / 28.3 l; *P* < 0.001). Seven cows showed mild ketonuria and another five cows showed moderate ketonuria. Mild metritis was diagnosed in five cows. The median duration of the surgery (excluding anaesthesia) was 44 min (42 min / 48 min; Q1 / Q3).

The length of the surgical wound (20.3 cm; 19.6 cm / 21.7 cm; median; Q1 / Q3) as well as the thickness of the everted sutured skin edges (1.8 cm; 1.7 cm / 1.9 cm) remained constant throughout the postoperative period.

Every wound healed *per primam* (primary intention healing) [17]. Slight soft swelling of the wound was observed in all cows at day 1 to 3 p. op. In most cases (62 %), the wound area was swollen ventrally. The wound area did not seem to be sensitive to palpation and was assessed as being warmer in comparison to the reference skin area in only 17 out of 140 palpation assessments (12 %).The wound area was never assessed palpatorically colder than the reference skin area.

In three cows, at days 1 and 2 p. op., slightly dried bloody outflow at the incision site was noticed. Beyond this, no exudation was observed. In four cows, a small skin patch was visible just on the first day after surgery, ventral to the transverse processes of the second lumbar vertebrae (LV). This temporary superficial skin change was not inside the RA outline.

The omentopexy incision was clinically normal in all cows but one where swelling was observed at days 9 and 10. The wound area above omentopexy site showed neither pressure pain nor remarkable temperature change through palpation. The ambient temperature (ϑ_Amb_) in the examinations room varied from 7.8 to 24.1 °C during the ten-month study period. Therefore, the cows were retrospectively classified into two groups based on the ambient temperature (ϑ_Amb_) using the Cluster Procedure with SAS® (Version 9.1, Statistical Analysis System Institute INC., Cary, NC, USA).

- Measurements at a relatively high ambient temperature (ϑ_Amb_) of 20.2 °C (18.5 °C / 21.7 °C; HT group; *n* = 6).
- Measurements at a relatively low ambient temperature (ϑ_Amb_) of 10.8 °C (9.4 °C / 12.8 °C; LT group; *n* = 8).

The median relative humidity in the examination room in the HT group was 67.4 % (60.0 % / 76.0 %) and in the LT group 75.5 % (70.0 % / 83.0 %).

The mean skin temperature in the reference area (ϑ_RA_) strongly correlated with the ambient temperature (*r* = 0.71; *P* < 0.001; Table 2), but only weakly with the rectal temperature (*r* = 0.28; *P* < 0.001). The preoperative ϑ_RA_ was lower compared to postoperative measurements (day1- day10) in the HT group (32.3 °C; 31.8 °C / 32.6 °C) as well as in the LT group (29.1 °C; 28.6 °C / 30.0 °C; *P* < 0.01). The ϑ_RA_ did not vary significantly in the postoperative period (between two different postoperative days). The median ϑ_RA_ in the postoperative period was 35.6 °C (35.0 °C / 36.1 °C) for cows in the HT group and 32.1 °C (30.9 °C / 33.0 °C) for cows in the LT Group.

**Table 2:** Spearman’s correlation coefficients between ambient temperature (ϑ_Amb_), rectal temperature and infrared (IR) thermographic measurements in 14 cows determined on the day of surgery and during the postoperative phase

Comparative parameters		r	P
Ambient temperature (ϑ_Amb_)	Mean skin temperature of the reference area using IR thermography (ϑ_RA_)	0.71	< 0.001
Ambient temperature (ϑ_Amb_)	Mean skin temperature of the wound area using IR thermography (ϑ_WA_)	0.69	< 0.001
Ambient temperature (ϑ_Amb_)	IR thermographic temperature difference Δϑ (ϑ_WA_-ϑ_RA_)	-0.51	< 0.001
Rectal temperature	Mean skin temperature of the reference area using IR thermography (ϑ_RA_)	0.28	< 0.001

The mean skin temperature of the wound area (ϑ_WA_) correlated with the ambient temperature (*r* = 0.69; *P* < 0.001; Table 2). In the postoperative period, the ϑ_WA_ was lower in both groups only at day 1 (d1) compared to other postoperative measurements (d2 - d10; *P* < 0.01).

The Δϑ was postoperatively higher for cows in the LT group (median of the individual animals 0.8–2.5 °C) than in the HT group (0.1–0.5 °C; *P* < 0.05). A negative correlation between Δϑ and ϑ_Amb_ was observed postoperatively (*r*=-0.51; *P* < 0.001; Table 2). The subjective impression of an elevated temperature (> 1 °C) in the wound area compared to the surrounding skin was given in 17 (12 %) of the total 140 manual palpations (Table 3). In these 17 wound assessments, which were palpatorically considered as warmer, Δϑ was 1.8 °C (1.1 °C / 2.1 °C). The Δϑ of the wounds (*n* = 123), which were not characterised by palpation as increased warmth, was significantly lower at 0.7 °C (0.2 °C / 2.0 °C; *P* < 0.05).

**Table 3:** Evaluation of the wound temperature by palpation in comparison to the skin temperature measurements with the infrared camera (IRT)

Number of measurements	HT group			LT group		
	palpatorically			palpatorically		
	positive	negative	total	positive	negative	total
IRT>1 °C	3	4	7	11	51	62
IRT<1 °C	2	51	53	1	17	18
total	5	55	60	12	68	80

LT: Group of cows whose measurements were taken at low ambient temperatures (*n* = 8; 80 measurements)

HT: Group of cows whose measurements were taken at high ambient temperatures (*n* = 6; 60 measurements)

**Fig. 3:**
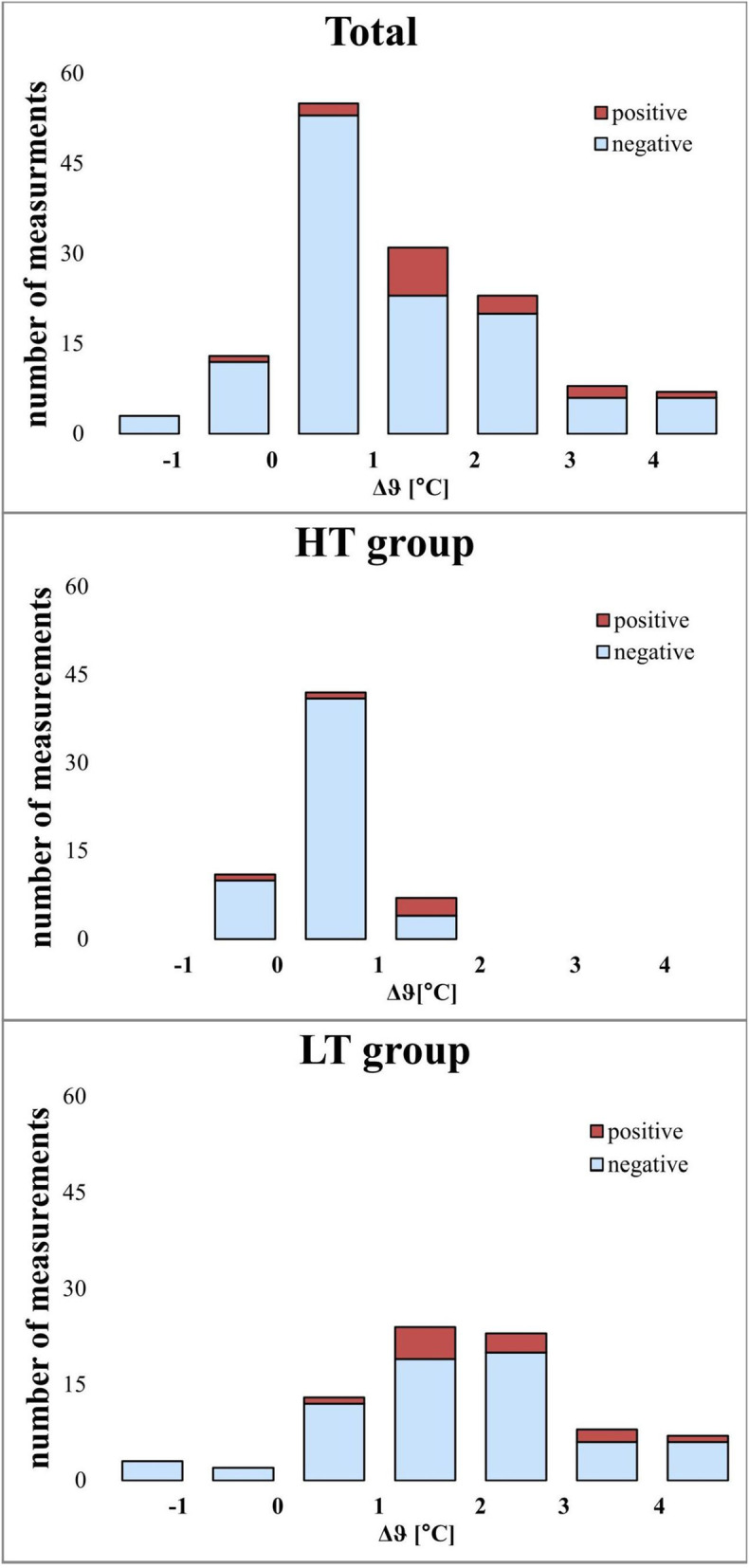
Distribution of thermographic temperature differences (Δϑ) for all cows and for both groups in detail. The shaded blue area of the columns indicates the cows diagnosed palpatorically showing no increased warmth; the shaded red area indicates those cows diagnosed showing increased warmth. Total: includes all the cows (*n* = 14; 140 measurements); LT: Group of cows whose measurements were taken at low ambient temperatures (*n* = 8; 80 measurements); HT: Group of cows whose measurements were taken at high ambient temperatures (*n* = 6; 60 measurements)

For the subjective manual assessment of Δϑ, a sensitivity of 20 % and a specificity of 96 % (for all cows in the study) were calculated. The assessment of skin temperature by palpation in cows in the HT group showed a significantly higher sensitivity (43 %) than in the LT group (18 %), and a very high specificity value was also calculated for the individual groups (Table 4). A total of 55 measurements (4/51; HT/LT) were false negative and three measurements (2/1) were false positive (Table 3). The exact distribution is shown in Fig. 3.

**Table 4:** Sensitivity (SEN) and specificity (SPC) of the assessment of wound temperature by palpation

	HT group	LT group	Total
SEN	0.43	0.18	0.20
SPC	0.96	0.94	0.96

LT: Group of cows whose measurements were taken at high ambient temperatures (*n* = 8; 80 measurements);

HT: Group of cows whose measurements were taken at high ambient temperatures (*n* = 6; 60 measurements)

## Discussion

IR thermography is a non-contact imaging method for measuring the thermal radiation of surfaces. This allows indirect estimation of blood flow changes and tissue metabolic activity [18]. Injuries or inflammation usually alter blood flow to affected tissues [19]. “Calor” (heat) as a cardinal symptom of inflammation is the result of increased blood flow [16]. Thus, a significant warming of a skin area may be an expression of inflammatory changes in the underlying tissues [20, 21]. In clinical practice, variations in skin temperature are checked by palpation during the clinical examination by using the dorsal surface of the examiner’s hand [4, 22]. The importance of hand hygiene palpating a surgical wound with ungloved hands should not be underestimated in order to minimise the risk of a nosocomial wound infection.

In this study, all the incisional wounds of the treated cows were covered with a chlortetracycline spray instead of an (adhesive) gauze dressing pad so as to avoid any skin irritation, which could have an impact on the thermographic measurements. In addition, the use of a micronised aluminum spray was not preferred, as it may cause reflections and could affect the IR measurements. All cows were administered procaine penicillin for four days, as this was the former standard procedure in our clinic at the time of the study. Taking into account the importance of prudent use of antibiotics, we would no longer suggest the perioperative use of antibiotics in non-complicated LDA surgery. The use of an antibiotic spray as wound protectant on incisions should also be avoided.

In this study, always the same person performed this examination at varying ambient temperatures and his hands were not warmed up. The subjectivity of sensory impressions in human medicine may impair the early detection of wound infections. Therefore, the regular clinical evaluation of wounds makes more sense if the examinations are consistently carried out by the same physician [23].

The practical use of IR thermography has been investigated in several fields of veterinary medicine and different animal species [24, 25]. In the present study, the sensitivity of the manual skin palpation of the wound area was very low compared to the objective thermographic measurements (20 %; Table 4). This result corresponds to findings in human medicine. Palpation is considered to be subjective and inaccurate. The temperature differences of approximately 2 °C are rarely detected with the hand in humans [5, 26]. IR thermography is considered ten times more sensitive for assessing temperature differences than manual palpation [16].For patients with a high risk of developing diabetic foot disease, a daily bilateral temperature control using an IR-thermometer is recommended. Temperature differences of more than 2.2 °C are considered critical and require further diagnostic evaluation [26]. In a study in human patients, four low cost IR thermometers were validated (under constant ambient conditions) assessing the skin surface temperature of the wound. All the thermometers were highly reliable (intraclass correlation coefficient greater than 95 %). Accordingly, a handheld and more cost-effective IR thermometer (pyrometer) seems to be an appropriate alternative to an IR camera for assessing the wound temperature reliably with contactless measurements [27].

According to a new study, the palpation detection of a warmer skin surface is possible but not always effective (only 62 % accuracy at Δϑ = 1 °C) despite strictly controlled environmental conditions. The efficiency rate depends on the temperature difference (Δϑ) as well as on the experience of the clinician [28].

Ambient temperature is one of the most relevant environmental factors influencing skin temperature [29, 30]. In human medicine, the impact of ambient temperature on skin temperature is of minor relevance, as the temperature in the examination room is less affected by seasonal changes. Thermographic measurements on large animals, on the other hand, are often difficult in practice due to the considerable temperature variation in the barn during the year. Similarly, Wilhelm et al. [31] noted that different ambient temperatures significantly impair the thermographic assessment of temperature differences between affected and healthy claws. Furthermore, Spire et al. [32] compared the surface temperature of beef cattle ears with or without steroids ear implants using an IR camera and noted that, thermoregulation in inflamed skin is different from normal skin. They also observed greater temperature differences in cases of cold and humid weather. Similar results were also reported in horses [33]. In the cited study, it was determined that the flank temperature was higher in pregnant than in non-pregnant mares. Ambient temperature influence on skin temperature was also observed. When ambient temperature was lower than 19 °C, a higher temperature difference was evident.

In the present study, higher differences in temperature between the wound area and regular skin were recorded with the IR camera at lower ambient temperatures in comparison to higher ambient temperatures. There were six measurements in the low temperature group where the calculated Δϑ was higher than 4 °C (4.1–5.1 °C; Fig. 3). In fact, these individual Δϑ occurred due to the lowest temperature values of the ϑ_RA_, which were determined on these days (27.2–30.6 °C) and not because of an extremely high absolute temperature measurement of the wound area (31.9–34.7 °C). These temperatures were noticed in different cows and always on a different day after surgery (d4-d10). Nevertheless, in our study, the sensitivity of the palpation assessment of skin temperature was significantly lower on colder than on warmer days (Table 4). This is probably due to the fact that the thermoreceptors of the (not warmed up) hands are less sensitive at lower ambient temperatures (approx. 11 °C) and may not perceive temperature differences of approx. 1 °C at a skin temperature of approx. 33 °C.

In the present study, the healing of the incisional wounds (*n* = 14) was closely monitored thermographically. The slight swelling, which occurred in all cows exclusively between days 1 and 3 p. op. and mostly only in the ventral third of the wound, is thus consistent with clinical unimpaired wound healing. The undisturbed general condition of all cows (no fever, good feed intake) and their increasing milk yield during the ten-day postoperative period confirm a clinical recovery after the surgery. The telephone interviews conducted with the owners 30 and 60 days after surgery did not highlight the presence of clinically detectable disturbances of wound healing. A higher number of cows would be desirable in principle to improve the statistical power of the results. On the other hand, the simultaneous inclusion of more cows in the study was only possible in exceptional cases due to the time-consuming diagnosis of the patients and the limited availability of pens and examination rooms. The fact that the ϑ_RA_ was lower in both groups preoperatively than on the days after surgery may indicate that the recently washed and shaved flank was not completely acclimatised to the ambient temperature.

In our clinic, the distal paraverterbral block combined with a linear infiltration of the incision line represents the established standard flank anaesthesia method. The additional use of the infiltration block prevents an incomplete anaesthetic result occurring on the incisional area when using only the distal paravertebral block, as the nerve pathways can vary [34]. Using epinephrine can prove advantageous, as bleeding from the incision during the surgery is minimal. On the other hand, epinephrine may also favor local tissue necrosis. The temporary superficial skin change under the transverse processes of the second LV shown in four cows was similar to that in the skin area where the syringe was injected with local anaesthetic. This may be associated with the vasoconstrictive effect of epinephrine. No significant differences however were found between the postoperative days.

There were no sudden or intense changes in ambient temperature during the study period. The mean temperature variation from one day to the other was only 0.6 °C. Rapid changes in ambient temperature (between days) would probably have made it more difficult to compare repeated measurements of skin temperature. Unanswered remains the question how the ambient temperature may affect the thermographic detection of a wound infection (the infection-induced warming of the skin). The present findings indicate that false negative diagnoses may occur in summer since the inflamed skin vessels are probably no longer proportionally dilatable as is the case in healthy skin. As a result, the entire flank appears warm. Furthermore, the short-term temperature increase in the wound area during the physiological healing process should not be considered as an incipient infection. This might lead to false positive diagnoses in winter. Therefore, additional studies should be carried out on cows with impaired wound healing.

## Conclusions

The palpation estimation of the skin temperature in bovine practice does not seem to be sufficient for a reliable temperature assessment of the wound area. As a limiting parameter, the ambient temperature influences the value of manual palpation. At low ambient temperatures in particular, the palpation sensitivity is further reduced. In contrast, the examination using the objective IR thermography is much more precise. However, it requires a higher technical and temporal effort. Future investigations using the smaller and more cost efficient pyrometer would be warranted.

## Data Availability

The datasets used and/or analysed during the current study are available from the corresponding author on reasonable request.
